# Frailty in hemodialysis and prediction of poor short-term outcome: mortality, hospitalization and visits to hospital emergency services

**DOI:** 10.1080/0886022X.2019.1628061

**Published:** 2019-06-25

**Authors:** Cesar Garcia-Canton, Ana Rodenas, Celia Lopez-Aperador, Yaiza Rivero, Gloria Anton, Tania Monzon, Noa Diaz, Nicanor Vega, Juan F. Loro, Angelo Santana, Noemi Esparza

**Affiliations:** a Department of Nephrology, Insular University Hospital of Gran Canaria, Gran Canaria, Spain;; b Faculty of Health Sciences, University of Las Palmas de Gran Canaria, Gran Canaria, Spain;; c Avericum Dialysis Center, Gran Canaria, Spain;; d Department of Nephrology, University Hospital of Gran Canaria Dr Negrin, Las Palmas, Spain;; e Faculty of Mathematics, University of Las Palmas de Gran Canaria, Gran Canaria, Spain

**Keywords:** Hemodialysis, frailty, outcome, mortality, hospitalization

## Abstract

**Background:** Frailty is an aging-associated state of increased vulnerability, which raises the risk of adverse outcomes. Chronic kidney disease is associated with higher prevalence of frailty. Our aim was to estimate frailty prevalence in a hemodialysis population and its influence on short-term outcomes.

**Design:** Observational prospective longitudinal study of 277 prevalent hemodialysis patients. Frailty was estimated through the Edmonton Frail Scale (EFS). Demographic and clinical data, comorbidity index, and laboratory parameters were recorded. A 29-month follow-up was conducted on mortality, including hospitalization, and visits to hospital emergency services in the first 12 months of this period.

**Results:** According to the EFS, 82 patients (29.6%) were frail, 53 (19.1%) were vulnerable, and 142 (51.3%) were non-frail. During follow-up, 58.5% frail patients, 30.2% vulnerable, and 16.2% non-frail ones died (*p* < .005). In the analysis of survival using an adjusted Cox model, a higher hazard of mortality was observed in frail than in non-frail patients (HR 2.34; 95% CI 1.39–3.95; *p* = .001). During follow-up the hospitalization rate was 852 episodes/1000 patient-years for frail patients, 784 episodes/1000 patient-years for vulnerable patients, and 417 episodes/1000 patient-years for non-frail patients (*p* = .0005). The incidence ratio of visits to emergency services was 3216, 1735, and 1545 visits/1000 patient-years for each group (*p* < .001).

**Conclusions:** Hemodialysis patients present high frailty prevalence. Frailty is associated with poor short-term outcomes and higher rates of mortality, visits to hospital emergency services, and hospitalization.

## Introduction

Frailty has been defined as a deterioration syndrome or state of increased vulnerability to stressful situations, resulting from aging-associated decline, characterized by a reduction in biological functional reserves, which raises the risk of poor outcomes such as the progression of disease, disability, hospitalization, and death [1–3]. Frailty is closely related to disability, dependence, and comorbidity, although these are different concepts, which do not always coexist [4].

Although frailty has been generally defined in association with advanced age, certain conditions that produce changes similar to aging may lead to a frailty state at younger ages. One of these conditions is chronic kidney disease [5,6]. Some studies have estimated 21–73% frailty prevalence in hemodialysis patients [7,8]. Such a large variability can be accounted for by differences in the studied populations or in the tools used to assess frailty. Tools have been classified into three types: those assessing the frailty phenotype through physical tests, subjective scales applied by healthcare staff and multi-domain scales evaluating further frailty dimensions such as cognitive, psychological, and social ones [9,10].

Some studies have established an association between frailty in hemodialysis and poor prognosis, leading to hospitalization, and death. In most of these studies, frailty was assessed through the presence of a frail phenotype as in the Fried Phenotype Frailty Index, or through subjective scales like the Canadian Frailty Scale [11–13]. However, there is less experience in the use of multi-domain scales, like the Edmonton Frail Scale (EFS), in hemodialysis.

Our objective was to estimate frailty prevalence in a hemodialysis population using the EFS [14] – a simple assessment tool comprising eleven items focusing on different frailty dimensions, which can be applied in the clinical practice – and to evaluate the association with demographic, clinical, and laboratory variables, as well as, with poor short-term outcome assessed through visits to hospital emergency services, hospitalization episodes, and death.

## Materials and methods

### Design

We conducted a prospective, observational, and longitudinal study with patients on the hemodialysis program in the South Healthcare Area of Gran Canaria, Spain, which corresponds to a population of 375 000. To estimate frailty prevalence, a cross-section was established for prevalent patients in October 2016. Inclusion criteria were: patient older than 18 years, more than 3-month prevalence in hemodialysis and ability to understand the information provided and to sign an informed consent form. Exclusion criteria were: patient with an active neoplastic or infectious disease or hospitalized in the previous 3-month period due to infectious, cardiovascular, or surgical complications. Demographic and clinical data were collected through the electronic medical records. Baseline complete laboratory tests were conducted on mid-week pre-dialysis blood extraction samples using the standard laboratory determination methods in our center. The modified Charlson comorbidity index [15,16] was calculated for all patients included in the study.

### Patients

Out of 294 available patients, 17 were excluded because they met the exclusion criteria, resulting in a final number of 277 patients included.

### Frailty

To estimate frailty prevalence, patients were administered the EFS, which comprises 11 items distributed into nine domains: cognitive (evaluated through the clock-drawing test), general health status, dependence, social support, medication, nutrition, depression, sphincter continence, and a physical test consisting of standing up and walking. Every item can be scored between 0 and 2, so that the global score varies from 0 to 17. Scores 0–5 correspond to non-frail, 6–7 to vulnerable, 8–9 to mild frail, 10–11 to moderate frail, and 12–17 to severe frail. For the statistical analysis, all frailty categories were grouped into frail: scores 8–17.

### Follow up

A one-year follow-up was conducted, where all visits to hospital emergency services were recorded through the electronic medical records. Additionally, all hospital admissions, their cause and duration, were recorded. A follow-up of patients was conducted until April 2019 (29 months), recording all deaths and any other reason for abandoning the study before the end of the follow-up period.

### Ethics

The study was conducted in accordance with the Declaration of Helsinki and was approved by the Clinical Research Ethics Committee in our Center. All patients signed written informed consent before participating in the study.

### Statistical analysis

Because the data did not follow a normal distribution, they were expressed as median and interquartile ranges (percentile 25 and 75, IQR) for continuous variables or as absolute frequency and percentages for qualitative variables. For comparison of qualitative variables between two or more groups, the Chi-squared test or the Fisher’s exact test were used, depending on data distribution. For comparison of continuous variables between two or more groups, the Mann–Whitney U-test or the Kruskal–Wallis test were used, as applicable. For all qualitative and continuous variables we also calculated the *p* values for the trend between groups using the appropriate test. All statistical tests were considered bilateral and significance was considered for *p* values lower than .05.

Survival times of patients under different frailty conditions were evaluated by a longitudinal cohort study. The Kaplan–Meier method was used to examine crude survival in the three groups defined by their frailty status (frail, vulnerable, and non-frail). Cox proportional hazards regression was applied firstly to estimate unadjusted hazard ratios (HRs) in the three groups. Next, multidimensional Cox regression was used to adjust for possible confounders. This was performed by entering all the variables potentially associated with survival into the model: Edmonton Frailty, age, sex, diabetes mellitus, months on dialysis, Charlson comorbidity index without age, body mass index (BMI), hemoglobin, albumin, prealbumin, T cholesterol, triglycerides, C reactive protein, uric acid, calcium, phosphate, intact parathyroid hormone (iPTH), creatine kinase, potassium, and creatinine. After using the backward selection method, only the significant variables remained: Edmonton frailty, Charlson comorbidity index without age, BMI, albumin, and creatine kinase. The Cox modeling results were summarized with HRs for each variable, 95% confidence intervals, and associated *p* values.

A negative binomial regression model was used to model both the number of hospital admissions and the number of emergency visits during the period of patient follow-up as a linear function of frailty status and other possible influential variables. This model was used as an alternative to Poisson regression model for count data due to its ability to adapt to situations in which the average number of events and their variance are different, as in this case. Because events were counted over different time intervals for different individuals, duration of the follow-up period was included in the model as an offset variable. Results of the negative binomial regression models were summarized as incidence rate ratios for each variable, 95% confidence interval and corresponding *p* values. As in the case of survival times, the negative binomial model was used first to estimate unadjusted incidence rate ratios for each frailty level (considering non-frail as the reference level), and then a stepwise selection method was used to include possible confounders in the model and calculate adjusted incidence rate ratios.

In all cases, hypothesis testing was considered significant when the corresponding *p* values were less than .05.

All statistical analyses were conducted with statistical software R version 3.5.3 (R Core Team (2019). R: A language and environment for statistical computing. R Foundation for Statistical Computing, Vienna, Austria. URL https://www.R-project.org/).

## Results

The study included 277 patients of 65 years median age (IQR 53–73): 182 men (65.7%) and 95 women (34.3%), with 34.6 months median time in dialysis (IQR 15.6–74.5); 159 patients were diabetic (57.4%). The most frequent etiology of renal disease was diabetic nephropathy (41.5%) followed by nephropathy of vascular origin (13%), interstitial nephropathy (8.3%), glomerular nephropathy (7.9%), ADPKD (7.9%), and nephropathy of other or unknown origin (the remaining 21.4%). The median Charlson index was 6 (IQR 5–8).

**Figure 1.**
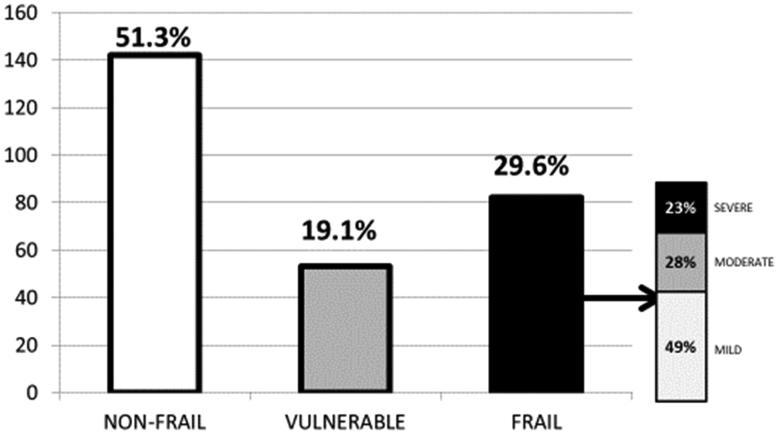
Prevalence of frailty among our hemodialysis population according to the Edmonton Frail Scale (EFS).

According to the EFS, 82 patients (29.6%) were frail, 53 patients (19.1%) were vulnerable, and 142 patients (51.3%) were non-frail. Among frail patients, 48.8% were mild frail, 28% were moderate frail, and 23.2% were severe frail. Figure 1 shows these results.

Table 1 shows demographic and laboratory variables for the different frailty groups. An association was found between frailty and certain variables often associated with poor prognosis, such as age, diabetes, high Charlson comorbidity index or female sex. Regarding laboratory parameters, we would like to highlight the association between frailty and lower hemoglobin, albumin, prealbumin, uric acid, phosphate, creatinine, and creatine kinase counts.

**Table 1.** Edmonton frail scale (EFS).

	Non-frail *N* = 142	Vulnerable *N* = 53	Frail *N* = 82	*p* Value for trend
Age	62 (50–69)	65 (54–76)	71 (63–78)	<.001
% Sex (male)	72.5	67.9	52.4	.003
% Diabetes	43	62.3	79.3	.001
Charlson comorbidity index	6 (4–7)	7 (4.5–9)	8 (6–10)	<.001
Months on dialysis	29 (15–67)	33 (17–78)	50 (21–82)	.047
Body mass index	27 (24–30)	27 (24–31)	26 (24–30)	.776
Hemoglobin, g/dL	11.7 (11–12.3)	11.3 (10.6–11.9)	11.1 (10.2–12)	.001
Glucose, mg/dL	109 (95–146)	114 (95–168)	138 (108–194)	.001
Albumin, g/dL	3.7 (3.4–3.8)	3.5 (3.3–3.8)	3.5 (3.2–3.8)	.001
Prealbumin, mg/dL	28 (24–33)	26 (21–31)	26 (20–30)	<.001
Uric acid, mg/dL	6.4 (5.4–7.3)	6.5 (5.3–7.1)	5.6 (5–6.3)	.001
Calcium, mg/dL	8.8 (8.2–9.3)	8.9 (8.5–9.2)	8.8 (8.4–9.3)	.550
Phosphate, mg/dL	4.4 (3.7–5.4)	4.5 (3.5–5.4)	4 (3.4–4.8)	.027
iPTH, pg/mL	277 (182–414)	225 (174–458)	272 (173–391)	.672
Creatin kinase, U/L	91 (58–143)	67 (50–107)	50 (29–82)	<.001
Creatinine, mg/dL	8.5 (6.4–10.4)	7.5 (6.1–9.7)	7.2 (5.6–8.3)	.001
T cholesterol, mg/dL	147 (121–175)	141 (124–156)	135 (112–163)	.033
Triglycerides, mg/dL	130 (92–191)	126 (95–181)	113 (80–181)	.121
Potassium, mM/L	5.3 (4.6–6.1)	5.5 (4.6–6.4)	5.3 (4.7–5.7)	.497
C reactive protein, mg/dL	0.41 (0.19–0.99)	0.61 (0.24–1.12)	0.48 (0.22–1.09)	.377

**Figure 2.**
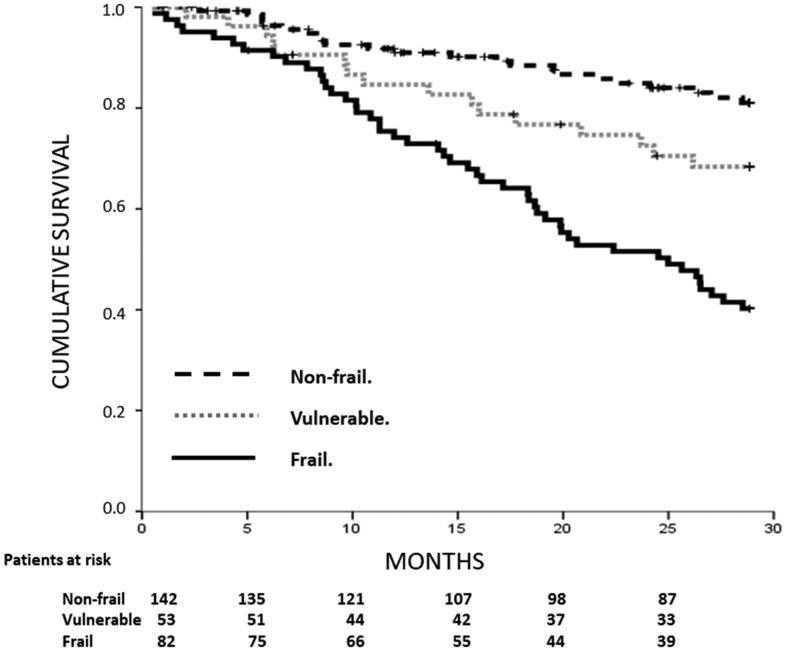
Kaplan–Meier survival curves by frailty status. Log-Rank test *p* values <.001.

**Figure 3.**
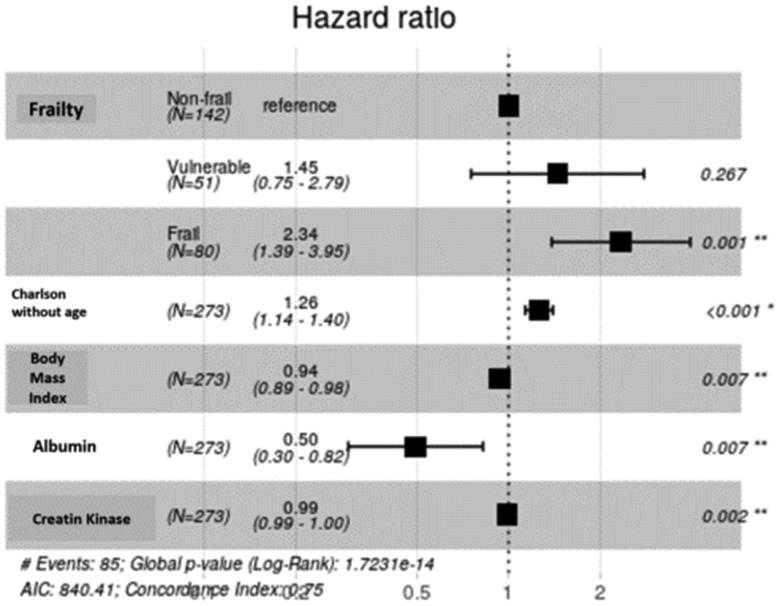
Multivariable Cox proportional hazards models of the association of frailty and mortality adjusted by Charlson Comorbidity Index without age, body mass index, serum albumin, and creatin kinase.

In the patient follow-up to April 2019, the mean follow-up time was 22 ± 9 months. In total, 145 patients (52.3%) completed the follow-up period, 87 (31.4%) died, 42 (15.2%) received a kidney transplant, and 3 (1.1%) were lost to follow-up after transferring to another medical center. The number of deaths was 48 patients in the frail group (58.5%), 16 in the vulnerable group (30.2%) and 23 in the non-frail group (16.2%). Figure 2 shows the Kaplan–Meier survival curves, with lower survival of frail than non-frail patients (*p* < .001). In the Cox model of patient survival, the unadjusted hazard rate of mortality for frailty, using the non-fragile group as a reference, was 1.8; 95% CI 0.94–3.5; *p* = .075 and 4.1; 95% CI 2.51–6.8; *p* < .001 for vulnerable and frail patients, respectively. When adjusted by the other significant variables for mortality, HR of frail compared to non-frail patients was 2.34; 95% CI 1.39–3.95; *p* = .001. Vulnerable patients did not show a significantly higher hazard than non-frail patients. The other significant variables maintained in the model were the Charlson index without age, BMI, albumin level, and CPK (Figure 3).

Excluding hospitalization for kidney transplant, the rates of hospitalization/1000 patient-years were 852, 784, and 417 episodes for frail, vulnerable and non-frail patients, respectively. To compare the incidence ratio of hospitalization between the three groups, a negative binomial regression model was used. Table 2 shows that the rate of hospitalization is significantly higher in the frail and vulnerable patients than in the non-frail patients, both in the unadjusted model and the model adjusted for other significant variables. Table 3 shows the data corresponding to hospitalizations in the three groups. It can be observed that the number of hospitalization days per patient and year, and the mean duration of hospital stay per hospitalization episode, were higher in frail patients. Regarding hospitalization causes, non-frail patients showed higher tendency to hospitalization due to complications with dialysis access and surgical interventions; while frail patients showed higher tendency to hospitalization due to infectious or cardiovascular complications. Additionally, bone fractures as the cause of hospitalization appeared in this group, but not in the others.

**Table 2.** Negative binomial regression hospitalization.

	IRR	Pr(>IzI)
**Unadjusted**		
(Intercept)	0.03 [0.03; 0.05]	*p* < .0001
Vulnerable	1.94 [1.20; 3.13]	.0056
Frail	2.09 [1.38; 3.18]	.0005
**Adjusted**		
(Intercept)	0.05 [0.01; 0.29]	.0007
Vulnerable	1.82 [1.13; 2.92]	.0124
Frail	1.78 [1.15; 2.77]	.0094
Charlson CI without age	1.16 [1.06; 1.27]	.0014
Albumin	0.61 [0.39; 0.96]	.0283
Phosphate	1.18 [1.03; 1.34]	.0100

**Table 3.** Hospitalization.

	Total *n* = 277	Non-frail *n* = 142	Vulnerable *n* = 53	Frail *n* = 82
%HP	37.9%	26.8%	41.5%	54.9%
H rate	615	417	784	852*
Days pat./y	5.8	3.2	6	10.1
ADS days	9.4	7.7	7.7	11.8
% Cause of admission				
Infectious	30.5%	24.1%	27.8%	37.5%
Cardiovascular	29.9%	24.1%	27.8%	35.9%
Dialysis access	14.9%	18.5%	13.8%	12.5%
Neoplasm	3.9%	7.4%	5.6%	0%
Bone fracture	2.0%	0%	0%	4.7%
Surgery	7.1%	16.6%	5.6%	0%
Other	11.7%	9.3%	19.4%	9.4%

%HP: percentage of patients admitted to hospital at least once during follow-up; H rate: hospitalization rate per 1000 patients-year; Days pat./y: number of days at hospital per patient and year; ADS days: average duration of stay in days

* *p*<.001.

The rates of visits to emergency services were 3216, 1735, and 1545 visits/1000 patient-years for frail, vulnerable and non-frail patients, respectively. To compare the rate of visits to hospital emergency services between the three groups, a univariate negative binomial regression model and a multivariate model adjusted for other significant explanatory variables were used.

Table 4 shows the results, in which the rate of visits to hospital emergency services among frail patients is almost twice that of non-frail patients and the difference is statistically significant after adjusting for other variables (*p* = .0002).

**Table 4.** Negative binomial regression emergency visits.

	IRR	Pr(>IzI)
**Unadjusted**		
(Intercept)	0.13 [0.10; 0.16]	*p* < .0001
Vulnerable	1.20 [0.80; 1.81]	.3744
Frail	2.20 [1.58; 3.08]	*p* < .0001
**Adjusted**		
(Intercept)	0.65 [0.13; 3.25]	.5716
Vulnerable	1.21 [0.80; 1.81]	.3541
Frail	1.91 [1.36; 2.70]	.0002
Charlson CI without age	1.11 [1.03; 1.20]	.0057
Albumin	0.55 [0.37; 0.80]	.0012
Uric acid	0.87 [0.79; 0.97]	.0087
Phosphate	1.20 [1.08; 1.34]	.0007

## Discussion

While there is no consensus on the definition of frailty, it is currently recognized that frailty is an age-associated status of reduced resilience and increased vulnerability to stressful situations, characterized by weakness and lower biological functional reserves, which entails higher risk of unfavorable outcomes toward disability, hospitalization, and death [1,2,17]. Although it is closely related to sarcopenia, dependence, comorbidity, and disability, and these situations influence each other, they do not always coexist [18].

Although frailty was initially defined as associated with aging, situations have been described, such as chronic kidney disease, which may not only increase its prevalence at advanced ages, but also result in earlier onset [5–7,19].

Large differences in the prevalence of frailty in hemodialysis patients found in previous studies may be due to several reasons, such as differences in the study populations in terms of age, morbidity, and inclusion of prevalent or incident patients. However, they may also be due to differences in the methods used to detect frailty. In general, three frailty-detection tests are used: those based on the description of a frail phenotype through physical tests, such as the one described by Fried [4]; those based on subjective assessment scales, such as the Frailty Score developed by Rockwood in Canada [20], and multi-domain tools that explore various frailty dimensions such as cognitive state, degree of dependence, psychological dimension, social support, or physical dimension, e.g., the Groningen Frailty Indicator [21], the Tilburg Frailty Indicator [22], and the EFS [14].

Most studies conducted on hemodialysis patients have used the Fried Frail Phenotype, observing frailty prevalence of 21.9–73% [11,12,23,24].

Studies on hemodialysis in which subjective scales were used to assess frailty described a prevalence of 19.6–26% [13,25].

Multi-domain tests assessing a range of frailty dimensions have been less studied in hemodialysis. Van Loon observed 67% frailty prevalence using the Groningen Frailty Indicator, although the study was conducted on an advanced-age population of 123 incident hemodialysis patients, all older than 65 years [26]. We have recently published our data for prevalent patients on hemodialysis measuring frailty using the Fried Phenotype Frailty Index and the EFS, where the proportion of frail patients was 41.2% when measured with the Fried criteria and 29.6% when measured with the EFS [27].

We chose the EFS because it can be easily administered in daily clinical practice and has been validated for different populations [9,28–30]. Using this test, we found 29.6% frailty prevalence in our population, which was close to the data reported with other methods in previous studies. Few studies have been published on the use of this test in hemodialysis patients. We found only two small studies: a study by Chao in Taiwan, including only 46 patients and reporting 43.6% frailty prevalence with the EFS, and a study by Orlandi, in Brazil, including 60 hemodialysis patients, all older than 60 years, reporting 38.3% frailty prevalence [25,31]. Our study is therefore the most extensive to date in which the EFS is applied to hemodialysis patients.

As shown in Table 1, frailty in hemodialysis patients is associated with demographic and clinical factors, which are usually associated with poor prognosis, such as older age, diabetes mellitus, and higher Charlson comorbidity index [32,33]. An association was also found between frailty and certain laboratory parameters such as lower hemoglobin, albumin, and prealbumin [34,35]. However, the association with laboratory parameters of bone and mineral metabolism and lipids is less clear. We failed to find an association with the inflammation marker C-reactive protein [36,37]. The association between frailty and serum creatinine and creatine kinase may be accounted for by lower muscle mass due to frailty-associated sarcopenia [38,39].

This relation found between surrogate markers of sarcopenia and frailty is of great interest. Sarcopenia is highly prevalent in hemodialysis and is associated not only with frailty, but also with the protein-energy wasting (PEW) syndrome. This syndrome, also known as malnutrition inflammation complex syndrome (MICS), is associated with poor prognosis [40,41]. We also found an association between frailty and markers of malnutrition (serum albumin and prealbumin). Further studies are needed to determine the relation between frailty and PEW in dialysis and its influence on prognosis.

Our results show a significant relation between frailty and mortality after a 29-month follow-up. In the Cox proportional hazards model, with the addition of other variables significantly related to mortality (age, diabetes mellitus, Charlson comorbidity index without age, and BMI) and analytical parameters associated with bone and mineral metabolism, lipid profile and nutrition parameters (albumin, prealbumin, creatinine, and CPK), the HR of mortality in frail patients compared to non-frail patients was 2.34; 95%CI 1.39–3.95; *p* = .001. These results agree with others previously published. In a study by McAdams-DeMarco on 146 hemodialysis prevalent patients, frailty was found to be a potent predictor of mortality at three years, when the risk was 2.6 times higher (CI 95%: 1.04–6.49) [12]. Johansen, in a study with 762 hemodialysis patients, observed a 2.1-fold higher risk of death at 2 years (CI 95%: 1.41–3.29) [11]. In both studies, frailty was assessed through the 5-item frailty phenotype described by Fried, including strength measured by a dynamometer and the walking speed test.

Two further studies with larger patient populations have been published: one by Bao on incident patients [24] and the other by Lee on prevalent patients [42]. In both studies, the Fried frail phenotype evaluated through questionnaires was used. Both studies showed independent association between frailty and mortality, with 1.57 (CI 95%: 1.25–1.97) and 2.37 (CI 95%: 1.11–5.02) HR in the first and second study, respectively. Alfaadhel evaluated frailty through the subjective clinical frailty scale in 390 incident hemodialysis patients. In this study, every incremental point on the frailty scale was associated with an increase in the mortality risk with a 1.22 HR (CI 95%: 1.04–1.43) [13]. Our results are in agreement with all of these studies. However, ours is the first study conducted on hemodialysis patients to show an association between mortality and frailty assessed through a multi-domain test that explores multiple aspects of frailty.

The number of hospitalization episodes has also been considered a sign of poor clinical prognosis, which may be associated with frailty. In our study, the percentage of patients admitted to hospital was significantly higher in frail and vulnerable patients than in non-frail patients. We also observed higher rates of hospitalization per 1000 patient-years among frail and vulnerable patients than among non-frail patients. In the cited studies by McAdams-DeMarco [12], Bao et al. [24], and Lee et al. [42], associations were demonstrated between frailty, number of hospitalization episodes, and time until first hospitalization. However, in those studies, frailty was assessed through the Fried Frailty Phenotype; while our study is the first to associate hospitalization with frailty assessed through a multi-domain test like the Edmonton test. Furthermore, in our study, a higher number of hospitalization episodes and longer mean hospital stay were found for frail patients. However, the mean stay duration was not adjusted for complexity and it might reflect a different patient profile, given that, as previously shown, frail patients are usually older, show higher mortality and their hospitalization causes tend to be different, e.g., cardiovascular and infectious causes are more frequent among frail patients, while vascular access complications and major surgery are more frequent among non-frail patients.

The number of visits to the emergency service is important information for the management of healthcare resources. It has been reported that failure to provide early primary care close to the patient’s home, in frail and multimorbid patients, impairs prevention of complications, and leading to frequent visits to hospital emergency services. This is important, because it may result in saturation of these services, thus preventing resource allocation to acute, potentially curable conditions, and increasing healthcare costs. Furthermore, the time frail patients spend in saturated emergency departments often results in healthcare-related adverse events, mainly of an infectious nature [43–45]. To the best of our knowledge, our study is the first to relate frailty in hemodialysis to the number of visits to hospital emergency services. We found that the rate of annual visits to emergency services in frail patients was twice that of non-frail patients. This difference was significant after adjusting for other variables in a negative binomial regression model. These findings are important because some studies have demonstrated that adequate frailty management through nutritional intervention strategies, physical exercise, and healthcare support, administered in the primary healthcare setting or in the dialysis units, may enhance patients’ baseline situation and eventually prevent poor short-term prognosis [46,47].

The EFS is very simple to administer and can be carried out by hemodialysis nursing staff after minimal training, without the need for specialist medical staff, in less than 15 min. Its association with poor short-term outcome highlights its utility in detecting frail patients, but because it is a multi-domain test, it also gives health professionals information about which areas of frailty are affected. This would allow specialists to administer specific, more complex scales to detect deficits in independence for basic and instrumental activities of daily life, cognition, emotional status, and social support. Detecting at-risk patients for a broader, more specific global geriatric assessment would allow health professionals to identify areas that require higher priority preventive or corrective action by the most appropriate professionals: geriatricians, neurologists, psychologists, physiotherapists, occupational therapists, and primary care and social work teams. The purpose of applying these preventive or corrective measures is to improve patient outcome and avoid complications associated with frailty.

The main strength of our study is that it is the first to analyze the association between frailty in hemodialysis patients, measured through a multi-domain test like the EFS, and poor clinical prognosis of patients. Additionally, we consider that including visits to hospital emergency services as a poor-prognosis variable gives greater interest to the study.

This study has also several limitations. First, it is a single-center study with a limited number of patients. It could also be argued that the EFS has not been validated in the general Spanish population, although many of the items used are commonly applied in Spain in other global geriatric assessment scales to measure dependence, depression, cognitive decline, and comorbidity. The EFS has also been used in a wide range of populations, including others in the Mediterranean that are similar to the Spanish population, e.g., Portuguese and Italian [28,48] and a Spanish version of the test validated in a Spanish speaking population in Colombia has been used [49]. Conducting the study on the prevalent but not the incident patient population may also be considered a limitation, because the frailty situation of patients at the beginning of renal replacement therapy was not known and may have been influenced by the time on therapy. Additionally, frailty was assessed at a particular time point, with no longitudinal follow-up, and therefore possible variations in the state of frailty and their impact on patient prognosis were not detected. However, a large impact on results is not expected, because this was a short-term follow up analysis. It would be interesting to compare the impact of frailty on patient outcome, assessed through a multi-domain test *versus* the frail phenotype, to determine whether including further frailty dimensions provides new data that could be useful for prognosis. For these reasons, the results of our study provide interesting data, although they should be taken with caution and supported by wider studies comparing the results of various frailty tests.

Our study demonstrated frailty prevalence in hemodialysis, assessed through the EFS, which was consistent with most of the studies where other test types were used. The results also showed that the occurrence of frailty, assessed through a multi-domain test was associated with poor outcome in hemodialysis patients, evidenced in higher mortality, hospitalization rates, and frequency of visits to hospital emergency services.

## References

[1] Clegg A, Young J, Iliffe S, et al. Frailty in elderly people. Lancet. 2013;381:752–762. 23395245 10.1016/S0140-6736(12)62167-9 PMC4098658

[2] Hogan DB, MacKnight C, Bergman H Models, definitions and criteria of frailty. Aging Clin Exp Res. 2003;15:1–29. 14580013

[3] Vermeiren S, Vella-Azzopardi R, Beckwée D Frailty and the prediction of negative health outcome: a meta-analysis. J Am Med Dir Assoc. 2016;17:1163e1–1163e17. 10.1016/j.jamda.2016.09.010 27886869

[4] Fried LP, Tangen CM, Walston J, et al. Frailty in older adults: evidence for a phenotype. J Gentorol Med Sci. 2001;56A:M146–M156. 10.1093/gerona/56.3.m146 11253156

[5] Reese PP, Cappola AR, Shults J, et al. Physical performance and frailty in chronic kidney disease. Am J Nephrol. 2013;38:307–315. 24107579 10.1159/000355568 PMC4019506

[6] Kim JC, Kalantar-Zadeh K, Kopple JD Frailty and protein-energy wasting in elderly patients with end stage kidney disease. J Am Soc Nephrol. 2013;24:337–351. 23264684 10.1681/ASN.2012010047

[7] Chowdhury R, Peel MN, Krosch M, et al. Frailty and chronic kidney disease: a systematic review. Arch Gerontol Geriatr. 2017;68:135–142. 27810661 10.1016/j.archger.2016.10.007

[8] Johansen KL, Chertow GM, Jin C, et al. Significance of frailty among dialysis patients. J Am Soc Nephrol. 2007;18:2960–2967. 17942958 10.1681/ASN.2007020221

[9] Dent E, Kowal P, Hoogendijk EO Frailty measurement in research and clinical practice: a review. Eur J Intern Med. 2016;31:3–10. 27039014 10.1016/j.ejim.2016.03.007

[10] Armstrong JJ, Stolee P, Hirdes JP, et al. Examining three frailty conceptualizations in their ability to predict negative outcomes for home-care clients. Age Ageing. 2010;39:755–758. 20858672 10.1093/ageing/afq121

[11] Johansen KL, Dalrymple LS, Glidden D, et al. Association of performance-based and self-reported function-based definitions of frailty with mortality among patients receiving hemodialysis. Clin J Am Soc Nephrol. 2016;11:626–632. 26792529 10.2215/CJN.03710415 PMC4822658

[12] McAdams-DeMarco MA, Law A, Salter ML, et al. Frailty as a novel predictor of mortality and hospitalization in individuals of all ages undergoing hemodialysis. J Am Geriatr Soc. 2013;61:896–901. 23711111 10.1111/jgs.12266 PMC3938084

[13] Alfaadhel TA, Soroka SD, Kiberd BA, et al. Frailty and mortality in dialysis: evaluation of a clinical frailty scale. Clin J Am Soc Nephrol. 2015;10:832–840. 25739851 10.2215/CJN.07760814 PMC4422241

[14] Rolfson D, Majumdar SR, Tsuyuki RT, et al. Validity and reliability of the Edmonton frail Scale. Age Ageing. 2006;35:526–529. 16757522 10.1093/ageing/afl041 PMC5955195

[15] Charlson ME, Szatrowski TP, Peterson J, et al. Validation of a combined comorbidity index. J Clin Epidemiol. 1994;47:1245–1251. 7722560 10.1016/0895-4356(94)90129-5

[16] Bedhu S, Bruns FJ, Saul M, et al. A simple comorbidity scale predicts clinical outcomes and costs in dialysis patients. Am J Med. 2000;108:609–613. 10856407 10.1016/s0002-9343(00)00371-5

[17] Morley JE, Vellas B, Abellan Van Kan G, et al Frailty consensus: a call to action. J Am Med Dir Assoc. 2013;14:392–397. 23764209 10.1016/j.jamda.2013.03.022 PMC4084863

[18] Rodriguez-Mañas L, Féart C, Mann G, et al. Searching for an operational definition of frailty: a Delphi method based consensus statement. The frailty operative definition-consensus conference project. J Gerontol A Biol Sci Med Sci. 2013;68:62–67. 22511289 10.1093/gerona/gls119 PMC3598366

[19] Wilhelm-Leen ER, Hall YN, Tamura MK, et al. Frailty and chronic kidney disease: the third national health and nutrition evaluation survey. Am J Med. 2009;122:664–671. 19559169 10.1016/j.amjmed.2009.01.026 PMC4117255

[20] Rockwood K, Song X, MacKnight C, et al. A global clinical measure of fitness and frailty in elderly people. CMAJ. 2005;173:489–495. 16129869 10.1503/cmaj.050051 PMC1188185

[21] Drubbel I, Bleijenberg N, Kranenburg G, et al. Identifying frailty: do the frailty index and Groningen frailty indicator cover different clinical perspectives? A cross sectional study. BMC Fam Pract. 2013;14:64–71. 23692735 10.1186/1471-2296-14-64 PMC3665587

[22] Gobbens RJJ, Van Assen MA, Luijkx KG, et al. The Tilburg frailty indicator: psychometric properties. J Am Med Dir Assoc. 2010;11:344–355. 20511102 10.1016/j.jamda.2009.11.003

[23] Takeuchi H, Uchida HA, Kakio Y, et al. The prevalence of frailty and its associated factors in Japanese hemodialysis patients. Aging Dis. 2018;9:172–207. 29896410 10.14336/AD.2017.0429 PMC5963342

[24] Bao Y, Dalrymple L, Chertow GM, et al. Frailty, dialysis initiation, and mortality in end-stage renal disease. Arch Intern Med. 2012;172:1071–1077. 22733312 10.1001/archinternmed.2012.3020 PMC4117243

[25] Chao C, Hsu Y, Chang P, et al. Simple self -report FRAIL scale might be more closely associated with dialysis complications than other frailty screening instruments in rural chronic dialysis patients. Nephrology. 2015;20:321–328. 25597434 10.1111/nep.12401

[26] Van Loon IN, Goto NA, Boereboom FT, et al. Frailty screening tools for elderly patients incident to dialysis. Clin J Am Soc Nephrol. 2017;12:1480–1488. 28716855 10.2215/CJN.11801116 PMC5586582

[27] García-Cantón C, Ródenas-Gálvez A, López-Aperador C, et al. Prevalencia de fragilidad y factores asociados en pacientes en programa de hemodiálisis. Nefrología. 2018;39:111–222 10.1016/j.nefro.2018.07.012. 30391021

[28] Perna S, Francis MD, Bologna C, et al. Performance of Edmonton Frail Scale on frailty assessment: its association with multi-dimensional geriatric conditions assessed with specific screening tools. BMC Geriatr. 2017;17:2. 28049443 10.1186/s12877-016-0382-3 PMC5209899

[29] Meyers BM, Al-Shamsi HO, Rask S, et al. Utility of the Edmonton Frail Scale in identifying frail elderly patients during treatment of colorectal cancer. J Gastrointest Oncol. 2017;8:32–38. 28280606 10.21037/jgo.2016.11.12 PMC5334059

[30] Partridge JS, Fuller M, Harari D, et al. Frailty and poor functional status are common in arterial vascular surgical patients and affect postoperative outcomes. Int J Surg. 2015;18:57–63. 25907322 10.1016/j.ijsu.2015.04.037

[31] Orlandi FS, Gesualdo GD Assessment of the frailty level of elderly people with chronic kidney disease undergoing hemodialysis. Acta Paul Enferm. 2014;27:29–34.

[32] Johnson JG, Gore SM, Firth J The effect of age, diabetes, and other comorbidity on the survival of patients on dialysis: a systematic quantitative overview of the literature. Nephrol Dial Transplant. 1999;14:2156–2164. 10489225 10.1093/ndt/14.9.2156

[33] Hecking M, Bieber BA, Ethier J, et al. Sex-specific differences in hemodialysis prevalence and practices and the male-to-female mortality rate: the dialysis outcomes and practice patterns study (DOPPS). PLoS Med. 2014;11:e1001750. 25350533 10.1371/journal.pmed.1001750 PMC4211675

[34] Robinson BM, Joffe MM, Berns JS, et al. Anemia and mortality in hemodialysis patients: accounting for morbidity and treatment variables updated over time. Kidney Int. 2005;68:2323–2330. 16221236 10.1111/j.1523-1755.2005.00693.x

[35] Gracia-Iguacel C, Gonzalez-Parra E, Perez-Gomez MV, et al. Prevalence of protein-energy wasting syndrome and its association with mortality in a haemodialysis patients in a centre in Spain. Nefrología. 2013;33:495–505. 23897181 10.3265/Nefrologia.pre2013.Apr.11979

[36] Iseki K, Tozawa M, Yoshi S, et al. Serum C-reactive protein (CRP) and risk of death in chronic dialysis patients. Neprol Dial Transplant. 1999;14:1956–1960. 10.1093/ndt/14.8.1956 10462277

[37] Lacson E, Levin NW C-reactive protein and end-stage renal disease. Semin Dial. 2004;17:438–448. 15660574 10.1111/j.0894-0959.2004.17604.x

[38] Ren H, Gong D, Jia F, et al Sarcopenia in patients undergoing maintenance hemodialysis: incidence rate, risk factors and its effect on survival risk. Ren Fail. 2016;38:364–371. 26738817 10.3109/0886022X.2015.1132173

[39] Lamarca F, Carrero JJ, Rodrigues JCD, et al. Prevalence of sarcopenia in elderly maintenance hemodialysis patients: the impact of different diagnostic criteria. J Nutr Health Aging. 2014;18:710–717. 25226111 10.1007/s12603-014-0505-5 PMC12880376

[40] Carrero JJ, Thomas F, Nagy K, et al. Global prevalence of protein-energy wasting in kidney disease: a meta-analysis of contemporary observational studies from the international society of renal nutrition and metabolism. J Ren Nutr. 2018;28:380–392. 30348259 10.1053/j.jrn.2018.08.006

[41] Kalantar-Zadeh K, Kopple JD, Block G, et al. A malnutrition-inflammation score is correlated with morbidity and mortality in maintenance hemodialysis patients. Am J Kidney Dis. 2001;38:1251–1263. 11728958 10.1053/ajkd.2001.29222

[42] Lee S, Yang DH, Hwang E, et al. The prevalence, association and clinical outcomes of frailty in maintenance dialysis patients. J Ren Nutr. 2017;27:106–112. 28065454 10.1053/j.jrn.2016.11.003

[43] George F, Evridiki K The effect of emergency department crowding on patient outcome. Health Sci J. 2015;9:6–12.

[44] Stang AS, Wingert AS, Hartling L, et al Adverse events related to emergency department care: a systematic review. PLoS One. 2013;8:e74214. 24069281 10.1371/journal.pone.0074214 PMC3772011

[45] Dent E, Hoogendijk EO, Cardona-Morrell M, et al. Frailty in emergency departments. Lancet. 2016; 387:434. 26869574 10.1016/S0140-6736(16)00177-X

[46] Tentori F, Elder SJ, Thumma J, et al. Physical exercise among participants in the Dialysis Outcomes and Practise Patterns Study (DOPPS): correlates and associated outcomes. Nephrol Dial Transplant. 2010;25:3050–3062. 20392706 10.1093/ndt/gfq138

[47] Fiatarone MA, O’Neill EF, Ryan ND, et al. Exercise training and nutritional supplementation for physical frailty in very elderly people. N Engl J Med. 1994;330:1769–1775. 8190152 10.1056/NEJM199406233302501

[48] Castro ML, Martins AM, Pinela AS, et al. Reproducibility of the adapted Portuguese Edmonton Frail Scale in elective cardiac surgery patients. J Cardiothorac Vasc Anesth. 2018;32:S42.

[49] Ramírez Ramírez JU, Cadena Sanabria MO, Ochoa ME Aplicación de la Escala de Fragilidad de Edmonton en población Colombiana. Comparación con los criterios de fried. Rev Esp Geriatr Gerontol. 2017;52:322–325. 28601218 10.1016/j.regg.2017.04.001

